# Normalising Choice: An Observational Study of Australian Clinicians' Perspectives on Written Informed Consent for Vaginal Birth

**DOI:** 10.1111/ajo.70061

**Published:** 2025-08-01

**Authors:** Harsha Ananthram, Venkat Vangaveti, Torres Wooley, Amy Dawes, Ajay Rane

**Affiliations:** ^1^ James Cook University Townsville Queensland Australia; ^2^ Wollongong Hospital Wollongong New South Wales Australia; ^3^ Birth Trauma Australia Buderim Queensland Australia

**Keywords:** caesarean section, information provision, informed consent, maternal choice, vaginal birth

## Abstract

**Background:**

The NSW Birth Trauma Report identified flawed consent processes and poor calibre antenatal information to have harmed birthing women. Written informed consent for vaginal birth may improve carer accountability and is currently applied in limited circumstances, for example, vaginal birth after caesarean section (VBAC).

**Aims:**

This study explores how informed women are about birth, as perceived by clinicians, and perspectives on the implications of written informed consent for vaginal birth.

**Materials and Methods:**

This study uses survey‐based research for quantitative data and inductive content analysis for open‐ended questions. Main outcome measures include carer perceptions on consent to the mode and/or location of birth and arguments against/in favour of written informed consent.

**Results:**

One thousand two hundred and seventy‐one responses were analysed for the final results, with 851 (67%) obstetric (Obs) and 420 (33%) midwifery (MW) respondents. Obs were eight times likelier to believe that women are never/rarely fully informed regarding vaginal birth (*p* < 0.001). The majority in both cohorts agreed women are frequently/always fully informed about VBAC. However, only 49 (6.6%) Obs and 20 (6%) MW were aware of written informed consent forms in use for vaginal birth. Themes developed include—‘helpless clinicians’ facing impediments to consent, flawed understanding of consent, rejection of consent requirements, juxtaposing consent with normality, disruption to collaboration and antenatal information undermining consent.

**Conclusions:**

Maternity carers in this Australian survey agree women are not fully informed regarding the risks and benefits of birth. Written informed consent alongside adjuncts like birth plans or technology‐based platforms may offer a way ahead for the future.

## 1 Introduction

The Birth Trauma Inquiry reports in New South Wales (‘NSW’) [1] and the United Kingdom (‘UK’) [2] have raised concerns about information provision and safety at birth. The NSW inquiry, which accepted submissions from women (over a period of two decades), makes several recommendations around consent [1]. This has been long overdue, as the absence of ‘formal birth preparedness/complication readiness’ [3] during antenatal care is a concern. A recent report from the UK [4] also highlighted, as an ‘essential action’, the need to ensure that ‘women have ready access to accurate information to enable their informed choice of intended place of birth and mode of birth, including maternal choice for caesarean delivery’.

The general law on consent does not require consent or the provision of information, including warnings about risks, to be in writing [5]. However, a written consent assists clinicians in providing appropriate and adequate information to patients in line with community expectations and legal requirements [5]. This article aims to enhance informed maternal choice at birth by analysing data and deriving themes from a national survey of maternity clinicians on their perceptions of informed consent. We evaluate the study results to understand why clinicians may wish to undertake written consent for vaginal birth and the arguments against it. We propose regulatory changes and solutions to improve consent standards. Critically, we emphasise how written consent may help cement the informal contract [3] between the carer and a woman. In this article, ‘woman’ represents all women and birthing people.

## 2 Materials and Methods

The online survey, in this study, used a combination of 31 closed‐ and open‐ended questions. The response options offered were in multiple‐choice or Likert scale formats as noted in Tables 1, 2, 3. Open‐ended questions were designed to elicit contextual insights into the quantitative data from close‐ended questions. The sample population of maternity clinicians included possibly 5000 respondents each, affiliated with the Royal Australian and New Zealand College of Obstetricians & Gynecologists (‘*RANZCOG*’) and the Australian College of Midwives (‘*ACM*’). Sample size calculations allowed for data from at least 257 participants in both respondent cohorts. This calculation determined the satisfaction percentage with a power of 0.90 and type 1 error of 0.05 (two‐tailed *t*‐test). The sample size was calculated using Open Epi, version 3.

**TABLE 1:** Informed birth choices.

To what extent, do you believe that women are *fully informed* about the benefits and risks of the mode and/or location of delivery in the following scenarios?	Response	Obstetric group (%)	Midwifery group (%)	Odds ratio	Confidence interval
Vaginal birth	Never/rarely	368 (49.9)	54 (16.3)	8.433***	5.922–12.010
	Sometimes	209 (28.4)	80 (24.1)	3.233***	2.321–4.503
	Frequently/always	160 (21.7)	198 (59.6)	Ref	
Vaginal birth after caesarean section	Never/rarely	36 (4.9)	38 (11.6)	0.332***	0.205–0.537
	Sometimes	74 (10)	69 (21.1)	0.376***	0.262–0.540
	Frequently/always	628 (85.1)	220 (67.3)	Ref	
Assisted vaginal birth	Never/rarely	174 (23.6)	109 (33.1)	0.474***	0.338–0.667
	Sometimes	277 (37.6)	135 (41.0)	0.610**	0.444–0.838
	Frequently/always	286 (38.8)	85 (25.8)	Ref	
Vaginal breech birth	Never/rarely	110 (16)	142 (46.7)	0.191***	0.138–0.263
	Sometimes	102 (14.8)	45 (14.8)	0.558**	0.372–0.837
	Frequently/always	475 (69.1)	117 (38.5)	Ref	
Water birth	Never/rarely	278 (47.4)	67 (24.1)	4.480***	3.147–6.378
	Sometimes	170 (29)	62 (22.3)	2.960***	2.041–4.293
	Frequently/always	138 (23.5)	149 (53.6)	Ref	
Home birth	Never/rarely	276 (52)	97 (45.5)	1.985***	1.392–2.831
	Sometimes	126 (23.7)	26 (12.2)	3.381***	2.049–5.578
	Frequently/always	129 (24.3)	90 (42.3)	Ref	
Caesarean section	Never/rarely	28 (3.8)	70 (21.3)	0.096***	0.060–0.154
	Sometimes	66 (9)	105 (32)	0.151***	0.106–0.215
	Frequently/always	639 (87.2)	153 (46.6)	Ref	

*Note:* Ref: the reference category is frequently/always. This parameter is set to zero because it is redundant. **p value < 0.01, ***p value < 0.001.

Data were analysed using statistical software IBM Corp (released 2023), IBM SPSS Statistics for Windows Version 29.0.2.0 Armonk, NY: IBM Corp. Continuous variables were tested for normality and based on the outcome of the test, parametric or non‐parametric analyses of the data will be undertaken. The chi‐squared or univariate binary/multinomial regression analysis was performed for determining associations between categorical variables. Multivariate regression model logistic regression helped determine factors leading to obtaining consent among health professionals. A *p* value of < 0.05 was considered statistically significant.

Data collected through the open‐ended questions were analysed by content analysis [6]. Inductive reasoning was used to derive themes that explain the collective understanding of the trends from survey respondents. In the first cycle, data described by the participants were initially distributed in responses related to informed consent and informed refusal. The second cycle of pattern coding involved visualisation maps with codes refined several times. This step specifically focused on responses related to informed consent. We derive six broad themes that help infer richer, more cohesive results from the quantitative data to construct the narrative of this article.

### 2.1 Ethics

The study is based on survey research with ethics approval from Townsville Hospital and Health Service Human Research Ethics Committee HREC/18/QTHS/88.

## 3 Results

Details relevant to the demographic questions in the survey, methods and results are previously published [7] and are not reproduced in this article. Of the 1271 responses (83% completion rate), analysed for the final results, 851 (67%) were from the obstetric group (‘*Obs*’) and 420 (33%) were from the midwifery group (‘*MW*’). Only 49 (6.6%) Obs and 20 (6%) MW respondents appeared to be aware of written informed consent forms for vaginal birth being used at their workplace.

Table 1 outlines to what extent, clinicians believe that women are *fully informed* about the benefits and risks of the mode and/or location of delivery. Nearly half of all Obs respondents believe women are never/rarely fully informed regarding vaginal birth. Obs were eight, four and approximately two times more likely than MW respondents to perceive that women are never/rarely fully informed regarding vaginal birth, water birth and home birth respectively. A large majority of respondents in both cohorts appear to agree that women are frequently/always fully informed about vaginal birth after caesarean (VBAC).

Table 2 outlines clinician perceptions on common arguments against written informed consent. Obs were twice as likely as MW respondents to believe written informed consent to be time‐consuming. MW respondents were approximately five times more likely than Obs respondents to perceive that written informed consent medicalises a ‘normal’ process and that it would increase intervention rates.

**TABLE 2:** Arguments *against* written informed consent for vaginal birth.

	Response	Obstetric group (%)	Midwifery group (%)	Odds ratio	Confidence interval
Time consuming	Not/minor importance	476 (64.3)	256 (79.5)	Ref	
	Important/very important	264 (35.7)	66 (20.5)	2.151***	1.579–2.932
Medicalises a ‘normal’ process	Not/minor importance	316 (42.6)	39 (12)	Ref	
	Important/very important	425 (57.4)	287 (88)	0.183***	0.127–0.263
Increases intervention	Not/minor importance	480 (64.9)	85 (26.6)	Ref	
	Important/very important	260 (35.1)	235 (73.4)	0.196***	0.147–0.262
Increases patient anxiety	Not/minor importance	219 (29.7)	35 (10.7)	Ref	
	Important/very important	519 (70.3)	291 (89.3)	0.285***	0.194–0.419
Vaginal birth is the ‘default’ option	Not/minor importance	316 (42.8)	70 (21.7)	Ref	
	Important/very important	422 (57.2)	252 (78.3)	0.371***	0.274–0.502

*Note:* Ref: the reference category is not important/minor importance. This parameter is set to zero because it is redundant. ***p value < 0.001.

Table 3 outlines clinician perceptions on common arguments favouring written informed consent. Obs are more likely than MW respondents to attach greater importance to all the parameters in favour of written informed consent, as noted in the survey.

**TABLE 3:** Arguments *in favour of* written informed consent for vaginal birth.

	Response	Obstetric group (%)	Midwifery group (%)	Odds ratio	Confidence interval
Medico‐legal reasons	Not/minor importance	212 (28.6)	198 (61.5)	Ref	
	Important/very important	528 (71.4)	124 (38.5)	3.977***	3.020–5.237
Standardise information provided	Not/minor importance	136 (18.4)	135 (42.2)	Ref	
	Important/very important	605 (81.6)	185 (57.8)	3.246***	2.430–4.336
Reduced decisional capacity in labour	Not/minor importance	210 (28.4)	156 (48.9)	Ref	
	Important/very important	529 (71.6)	163 (51.1)	2.411***	1.838–1.363
Reduced decisional conflict regarding mode or location of delivery	Not/minor importance	213 (28.9)	152 (47.6)	Ref	
	Important/very important	524 (71.1)	167 (52.4)	2.239***	1.707–2.937

*Note:* Ref: The reference category is not important/minor importance. This parameter is set to zero because it is redundant. ***p value < 0.001.

Table 4 outlines the themes derived from an inductive analysis of open‐ended question responses. Examples of participant comments organised by derived themes are presented in this table.

**TABLE 4:** Identified themes and clinician comments.

**1. The helpless clinician**
‘How is it that we consent women for caesarean and VBAC and don't tell them about the risks of a vaginal delivery?’
‘…I put to Executive 10 years ago that we should consider developing a consent form for vaginal delivery, and they laughed at me. A month later the hospital got sued for a 4th degree tear from a vaginal delivery…’
‘It's no longer a secret about the risks women face with an attempted vaginal birth. It seems to me that we often dissuade women from an elective caesarean and yet are shocked when the nulliparous woman needs an assisted vaginal delivery and is shocked by the outcome’
‘…If we emphasise the risks we are “scaremongering” and being negative about a natural process’
‘The politics of childbirth make informed discussions almost impossible. It is crazy that the most dangerous thing that a woman and child are likely to attempt is not discussed in detail’
**2. Conceptual flaws around consent**
‘I think the decision as to whether a birth is vaginal or otherwise should be dependent on the medical circumstances and not personal choice in the absence of good clinical/psychological indications’
‘Standardising information does not allow for individualised woman‐centred care—listen to what she says she needs!’
‘…informed consent, in private obstetric care can be inconsistent and dependent on individual management styles, the particular preferences of the obstetrician and their routine practices’
‘These documents are lazy medicine. They are being increasingly used to truncate a consultation, as a weapon and because of poor communication skills’
‘It is impossible within a timely manner to inform a woman of every possible complication that could happen, and therefore, the consent forms would be worthless’
‘That, things have come to the point where a consent form for labour and vaginal birth is being considered is in and of itself representative of how birth has come to be viewed, how it is represented and how it is thought of. How telling’
**3. Rejection of the requirement for consent**
‘…creates vaginal birth as an “option” which it is not, it is the logical result of pregnancy’
‘Consent to birth in the way the body was designed to do in most circumstances is beyond ridiculous’
‘…A form to sign to consent to vaginal birth is a public health disgrace. Make women sign forms to consent to pregnancy?’
‘However getting “consent” for a vaginal delivery is treacherous, as it adds great confusion for women, I believe. Women expect that if something is discussed as “consent”, then there is the option to choose something else’
‘…This is within the scope of practice of midwifery and in my professional opinion I do not believe women need to sign a medicolegal document stating they consent to giving birth in the biological intended way’
**4. Juxtaposing consent with normality of vaginal birth**
‘…When women enrol in a midwifery model of care which has evidence for improved outcomes for women, that comes with an understanding of desire for support towards normal birth’
‘…the push towards normal birth has led to an increase in the rate of instrumental delivery (especially forceps) with subsequent morbidity. I get the feeling that many of my colleagues feel that once a woman gets to full dilatation, vaginal birth must be attempted even with malposition or relatively high station’
‘Outcomes of women and their babies under a continuity model of care are far superior than standard care. Women under standard care often opt for epidurals and need intervention; which in turn has poor outcomes even if they have a vaginal birth…’
‘Informed consent for vaginal birth would cause the caesarean section rate to blow out’
**5. Lack of collaboration in information provision**
‘There can be no informed consent when coercion and bullying tactics are used, which is every time a Dr speaks to a woman! The dead baby card is played every time. This is blatant coercion’
‘…what I say is often trumped by the doctor in highlighting risks over benefits in many circumstances’
‘The arrival of purist direct entry midwifery training has increased the conflict and biased information in our area. It has not helped collaboration’
‘Have felt an increasing culture of irrational fixation on vaginal birth at all costs. Particularly via less experienced midwives, new graduates. Medical participation into patients antenatal education is actively resisted’
‘It appears that medical staff (often for medico‐legal reasons) feel that midwifery staff cannot understand or appropriately inform women of potential outcomes, even when practising for many years’
**6. Antenatal information undermining informed consent**
‘Patients are not given a balanced orientation of the potential difficulties of pregnancy and delivery during their prenatal care so that a false expectation is given’
‘I think a great deal of the conflict and angst that exists when we enter the room to offer assistance with birth via forceps, in particular, could be allayed by thorough counselling from the antenatal clinic appointments. Why is it such a secret? It seems so paternalistic to me. Just tell women the truth and let them make an informed decision’
‘An informed consent for instrumental delivery should be taken antenatally, not in an emergency situation, when weighing the risks and benefits could be difficult’
‘Current birth education is written and approved by midwives, therefore is understandably biased and pro “normal”, but brings to question whether current education actually provides “informed” consent’

## 4 Discussion

This research suggests that many clinicians agree women are not fully informed regarding vaginal birth, but lack interdisciplinary consensus on whether written consent is a solution. We believe that these results complement the consumer submissions to the birth trauma inquiry [1] and highlight how clinical practice is rife with arbitrary inconsistencies. For example, a perineal repair undertaken in a birth unit is often based on verbal consent, but the same procedure in an operating theatre reflexively captures written consent. Consider this discrepancy: Research participants in this study were mandated to document voluntary, free and informed consent more rigorously than women who give birth vaginally. One submission [1] to the inquiry captures this sentiment well:

It is the more insidious features of everyday practice within the hospital system that I believe contributes to the majority of birth trauma. This includes women lacking a sense of agency when navigating the maternity system, and the frequent absence of genuine choice, informed consent and individualised care. Without these, women cannot leave hospital feeling that they had an empowering experience.

### 4.1 Why Written Consent?

Consent for vaginal birth is best done during the antenatal period. Studies have shown that women self‐identify as possessing reduced decisional capacity during labour [3]. Birth Trauma Australia (‘*BTA*’) submits [1] that a ‘lack of proactive communication’ can lead women to feel ‘blindsided’ by their birth experience and unable to advocate for themselves effectively. Written consent, on paper, remains the most cost‐efficient means of documentation [8] and benefits both women and clinicians. There is less scope for clinician bias [7] corrupting written consent. Documented consent is particularly useful in determining legal disputes over facts. Courts prefer the ‘reliability and veracity of clinical notes over witness statements’ [9] when the woman and clinician differ starkly on what transpired during a consultation.

In many maternity units, women seeking a VBAC—for emphasis, *vaginal* birth—sign a form to ‘give’ consent. It is noted (Table 1) that both Obs and MW respondents in the survey broadly believe that consent works well for women counselled on VBAC. Why is this so? We claim that health organisations accord VBAC the serious consideration that vaginal birth often fails to attract. Organisations confront consumer advocacy for choice, service capability issues and interdisciplinary collaboration to ensure safe VBAC‐friendly service provision. The use of validated predictive tools helps personalise care for these women [10]. We ask whether written consent processes can effectively work with spontaneous vaginal birth too?

Women need protection from normality‐centred care [7] particularly when the focus on normality denies women choices that might reduce harm from vaginal birth. A disturbing theme emerges (Table 4) where some clinicians' views on normality, render them hostile to maternal choice. The Montgomery judgement [11], it is worth reminding, is uncompromising on choice:

A patient is entitled to take into account her own values, her own assessment of the comparative merits of giving birth in the “natural” and traditional way and of giving birth by caesarean section, whatever medical opinion may say, alongside the medical evaluation of the risks to herself and her baby.

Maternal choice matters. The BTA says ‘we need to stop infantilising women and we need to actually empower women with information’ [1]. It is absurd to presume that women with increased knowledge will likely opt for a caesarean section (‘*CS*’) [12]. Studies on information provision with risk stratification have shown remarkably low CS preference rates [13]. CS rates are no longer—and Australia would do well to take note—useful as a marker of the quality of maternity care [4]. We aspire to see care move from ‘normal birth’ to normalising maternal choice at birth.

Written consent for vaginal birth *enforces* choice. Some argue that [14] clinicians must go beyond passive acquiescence to maternal requests for CS (‘*MRCS*’). They ‘submit that healthcare practitioners caring for women with uncomplicated pregnancies have a positive duty to inform them of the option’ of MRCS and not just divulge this information when requested by the patient. Standardising counselling to bring this into practice is not cumbersome. RANZCOG provides updated evidence [15] on choosing between vaginal birth and a CS, that can guide counselling broadly.

### 4.2 Why Not?

A traditional view lingers (Table 4) that clinicians do not need to obtain consent for vaginal birth, an ‘inevitable physiological process’ [16]. A paradoxical proposal helps challenge this view. A woman can decline to give verbal consent to labour before birth. On a technicality, each case of MRCS represents a refusal to give birth vaginally. Clinicians support this [15] because how a woman chooses to give birth is a preference‐sensitive decision. Why is this choice not extended to all pregnant women? If published data [17] suggest that interventions are more readily available to women ‘of Australian origin’ or ‘of higher socioeconomic status’ the question begs to be asked; what of the women who do not share this privilege? The idea that vaginal birth is a default option, is no longer tenable.

The notion that written consent for vaginal birth makes women anxious is paternalistic. Some have argued that information may even assist such anxiety [18]. Perceptions of risk vary among consumers, midwives, doctors and hospital executives. Discussing risk demands nuance from the clinician. An ‘informal contract’ [3] exists between the carer and the woman giving birth at a hospital. Clinicians are duty‐bound to ensure that the reasonably foreseeable effects [18] of harm from birth are made explicitly clear. A written consent reflects, in document form, such a discussion.

### 4.3 Regulation & Solutions

Addressing consent‐related medicolegal claims [19] incentivises organisational change. Accountability for inadequate consent at birth rests with the clinician, organisation and regulatory processes. It could be argued that the ACM's stance on risk‐based regulation (creating restrictions for midwives) [20] effectively de‐risks pregnancy. A bias against complexity [21] is neither safe nor woman‐centred. The birth trauma reports have reinforced this. Managing risk encompasses themes as diverse as informed consent, clinician education, protocol development and documentation [22]. Organisations cannot ignore consumer expectations for a formal informed consent process to be introduced at birth [3].

Mandating written consent also assists clinicians who struggle conceptually with consent (Table 4). This theme is mirrored in the inquiry report [1] where the Maternity Consumer Network states ‘that despite the legal and ethical imperative for informed consent, maternity care providers often have a poor understanding of their legal responsibilities, which can further exacerbate problems’. Change within organisations involves staff education, audits and performance standards being established/met around consent documentation.

Organisations still place the onus of consent on the medical practitioner. Not all women need or can access obstetric clinicians during pregnancy. We argue that midwives, especially those in continuity models of care, inherit primary legal accountability for consent standards at birth. Courts place greater emphasis on adequate consent than on who provides this information. Succinctly put:

What is required is that the patient have the sufficient material in order to make an informed consent. It really does not matter where that information comes from. [23]

Such a proposal assumes basic expectations. First, this does not preclude women from seeking obstetric care as and when required/sought. Second, professional boards ensure that clinician scope of practice standards unambiguously address consent obligations at birth. Third, Obstetric & Midwifery Colleges publish collaborative documents on informed consent standards that clinicians adhere to and are held accountable for.

We challenge traditional perceptions of what is accepted as written consent. Consent modelled on birth plans may be an idea whose time has arrived. Birth plans are adjuncts to consent forms. A birth plan formulated by a woman is no less of a contract that binds the therapeutic relationship, tailored to the womans individual needs. It allows clinicians to dissect, in granular detail, concerns regarding care outside the domain of medical recommendations. Hearteningly, organisations have developed policies supportive of requests from women declining care [24]. It is time to extend this ‘generosity’ of choice to all pregnant women.

Consent is already moving beyond the realm of paper, much as womens expectations of consent are fast outpacing organisational change. Mobile artificial intelligence (‘*AI*’) apps can generate recordings of consultations. These apps are readily accessible to women. These AI recordings are, to use the legal term, ‘discoverable’ documents in a medicolegal claim. We believe such recordings will likely supplant consent forms—in all health disciplines—soon. Dynamic consent (‘*DC*’) in obstetric practice promises transformational change [25]. The use of technological platforms on mobile phones, integrated with adaptive technology, can make information accessible to women across the spectrum of health literacy. Women value ongoing discussions in pregnancy about the preferred mode of delivery [12] that DC can accommodate. DC will also allow for consent traceability and documentation throughout this time [8].

### 4.4 Limitations

We advise caution with the interpretation of results from this observational study. Even though the survey was conducted in 2018, it holds value when contextualised in the chronology of inquiry submissions spanning nearly two decades. We reiterate that the results of this survey have presaged what the birth trauma reports from both the UK and NSW now confirm. Women are clearly saying that current standards for consent at birth are unacceptable.

Overall, this study supports the principal finding in the NSW report [1] that ‘prospective parents need to be provided with clear and comprehensive education about all aspects of pregnancy and childbirth so that consent given to any obstetric intervention is fully informed’. A woman's pregnancy journey can take ‘many forking paths’ [18] with multiple possible outcomes, including severe complications that may ensue. De‐risking pregnancy by withholding information does not help. Clinicians must endeavour to ensure that a pregnant woman is aware of the harms and benefits of the alternative ways in which she may *choose* to give birth. Information provision demands ideology‐proofing to protect consumers from fluctuating and conflicted advice on birth. Written informed consent for vaginal birth is a solution worth considering.

## Conflicts of Interest

The authors declare no conflicts of interest.

## Data Availability

The data that support the findings of this study are available from the corresponding author upon reasonable request.

## References

[1] Select Committee on Birth Trauma , Birth Trauma (New South Wales Parliament Legislative Council, 2024), Report Number 1, https://www.parliament.nsw.gov.au/tp/files/188640/Report%20No%201%20‐%20Birth%20Trauma%20‐%20Tabled%2029%20May%202024.pdf.

[2] K. Thomas and The All‐Party Parliamentary Group on Birth Trauma , Listen to Mums—Ending the Postcode Lottery on Perinatal Care (UK Parliament, 2024), https://www.theo‐clarke.org.uk/files/2024‐05/Birth%20Trauma%20Inquiry%20Report%20for%20Publication_May13_2024.pdf.

[3] S. Ely , S. Langer , and H. P. Dietz , “Informed Consent and Birth Preparedness/Complication Readiness: A Qualitative Study at Two Tertiary Maternity Units,” Australian and New Zealand Journal of Obstetrics and Gynaecology 62, no. 1 (2022): 47–54, 10.1111/ajo.13417. 34455584

[4] D. Ockenden , “Findings, Conclusions and Essential Actions From the Independent Review of Maternity Services at The Shrewsbury and Telford Hospital NHS Trust,” House of Commons. Our Final Report, accessed April 05, 2025, https://assets.publishing.service.gov.uk/media/624332fe8fa8f527744f0615/Final‐Ockenden‐Report‐web‐accessible.pdf.

[5] NSW Ministry of Health , Consent to Medical and Healthcare Treatment Manual (Policy and Procedure Manuals/NSW Health, 2020), Updated July 2023, accessed June 05, 2025, https://www.health.nsw.gov.au/policies/manuals/Pages/consent‐manual.aspx.

[6] J. Saldaña , “The Coding Manual for Qualitative Researchers,” 2021.

[7] H. Ananthram , V. Vangaveti , and A. Rane , “Have We Lost Sight of the Women? An Observational Study About Normality‐Centred Care in Australian Maternity Services,” Australian and New Zealand Journal of Obstetrics and Gynaecology 62, no. 1 (2022): 40–46, 10.1111/ajo.13462. 34841509

[8] A. M. Tassé and E. Kirby , “Is Written Informed Consent Outdated?,” European Journal of Public Health 27, no. 2 (2017): 195–196, 10.1093/eurpub/ckw197. 27815285

[9] E. Ferriman , E. Power , and S. Jha , “Caesarean Section Including Uterine Rupture and Full Dilatation Caesarean Deliveries,” in Lessons From Medicolegal Cases in Obstetrics and Gynaecology (Cambridge University Press, 2022), 165–178, 10.1017/9781108993388.015.

[10] A. Gunaseelan , G. Cruz , Y. Y. El‐Sayed , C. Johnson , and Y. J. Blumenfeld , “Comparison Between the Old and New MFMU TOLAC Calculator—Which Is More Accurate?,” American Journal of Obstetrics and Gynecology 226, no. 1 (2022): S394, 10.1016/j.ajog.2021.11.659.

[11] “Moentgomery v Lanarkshire Health Board [2015] UKSC 11”.

[12] J. Bihler , R. Tunn , C. Reisenauer , et al., “The Preferred Mode of Delivery of Medical Professionals and Non‐Medical Professional Mothers‐To‐Be and the Impact of Additional Information on Their Decision: An Online Questionnaire Cohort Study,” Archives of Gynecology and Obstetrics 299, no. 2 (2019): 371–384, 10.1007/s00404-018-4970-7. 30467635

[13] D. Wilson , J. Dornan , I. Milsom , and R. Freeman , “UR‐CHOICE: Can We Provide Mothers‐To‐Be With Information About the Risk of Future Pelvic Floor Dysfunction?,” International Urogynecology Journal 25, no. 11 (2014): 1449–1452, 10.1007/s00192-014-2376-z. 24740445

[14] R. C. Brown and A. Mulligan , “‘Maternal Request’ Caesarean Sections and Medical Necessity,” Clinical Ethics 18, no. 3 (2023): 312–320, 10.1177/14777509231183365. 37635933 PMC7614977

[15] Guideline Development Group , “Caesarean Birth on Maternal Request (CBMR) (C‐Obs 39). Statements and Guidelines Directory/RANZCOG,” 2010, Updated July 2023, accessed April 05, 2025, https://ranzcog.edu.au/wp‐content/uploads/Caesarean‐Birth‐Maternal‐Request.pdf.

[16] S. Downe , “Informed Consent Should Be Obtained Before Vaginal Birth: AGAINST: Informed Consent Should Not Be Obtained Before Vaginal Birth: AGAINST: Informed Consent Should Not Be Obtained Before Vaginal Birth,” BJOG: An International Journal of Obstetrics & Gynaecology 129, no. 5 (2022): 830, 10.1111/1471-0528.15934. 35315577

[17] H. G. Dahlen , C. Thornton , S. Downe , et al., “Intrapartum Interventions and Outcomes for Women and Children Following Induction of Labour at Term in Uncomplicated Pregnancies: A 16‐Year Population‐Based Linked Data Study,” BMJ Open 11, no. 6 (2021): e047040, 10.1136/bmjopen-2020-047040. PMC8169493 34059509

[18] K. Irvine , R. C. Brown , and J. Savulescu , “Disclosure and Consent: Ensuring the Ethical Provision of Information Regarding Childbirth,” Journal of Medical Ethics 51, no. 8 (2023): 550−557, 10.1136/jme-2022-108283. PMC12322403 37308280

[19] J. Badenoch , E. Power , and S. Jha , “Consent: Legal and Clinical Implications,” in Lessons From Medicolegal Cases in Obstetrics and Gynaecology (Cambridge University Press, 2022), 41–54, 10.1017/9781108993388.006.

[20] A. Weatherstone and H. White , Unleashing the Potential of Our Health Workforce (Scope of Practice Review) (Australian College of Midwives, 2024), https://midwives.org.au/common/Uploaded%20files/ACM%20Submission_SoP_Mar_2024_final%20(2).pdf.

[21] M. Knight and C. Bevan , “Achieving Safer Maternity Care in the UK,” BMJ 372 (2021): n45, 10.1136/bmj.n45. 33436374

[22] L. A. Miller , “Water, Water Everywhere … or Perhaps Not? Informed Consent and Risk Management for Underwater Birth,” Journal of Perinatal and Neonatal Nursing 28, no. 3 (2014): 164–166, 10.1097/JPN.0000000000000045. 25062516

[23] “Olbourne v Wolf [2004] NSWCA 14”.

[24] Working Group , Guideline: Partnering With the Woman Who Declines Recommended Maternity Care (Clinical Excellence Queensland/Queensland Health, 2025), https://www.health.qld.gov.au/__data/assets/pdf_file/0022/736213/maternity‐decline‐guide.pdf. 10.1111/ajo.13889 PMC12099174 39333011

[25] H. Ananthram and A. Rane , “‘Petrificus Totalus’: Dynamic Consent in Obstetric Practice?,” BJOG: An International Journal of Obstetrics & Gynaecology 129, no. 12 (2022): 1957–1960, 10.1111/1471-0528.17259. 35856877 PMC9796783

